# HPK1 kinase inhibitor: a sufficient approach to target HPK1 to modulate T cell activation in cancer immunotherapy compared with degraders

**DOI:** 10.3389/fimmu.2025.1449106

**Published:** 2025-02-06

**Authors:** Qin Wang, Xinyi Zhu, Jing Li, Sanjia Xu, Ali Wang, Xinwen Zhang, Xingxing Wang, Xiaopeng Cai, Haimei Xing, Ye Liu, Xuesong Liu, Zhiwei Wang, Lai Wang, Xi Yuan

**Affiliations:** ^1^ Department of Biology, BeiGene (Beijing) Co., Ltd., Beijing, China; ^2^ Department of Medicinal Chemistry, BeiGene (Beijing) Co., Ltd., Beijing, China; ^3^ Research & Clinical Development, BeiGene (Beijing) Co., Ltd., Beijing, China

**Keywords:** hematopoietic progenitor kinase 1, kinase, non-catalytic function, T cell receptor signaling, degrader

## Abstract

**Background:**

Hematopoietic progenitor kinase 1 (HPK1) is a member of the mitogen-activated protein kinase kinase kinase kinase (MAP4K) family. It has been reported that HPK1 negatively regulates the activation of T cells. Several compounds have been developed and tested in clinical trials to target HPK1 for cancer immunotherapy. However, whether kinase inhibition is sufficient to eliminate the immunosuppressive function of HPK1, particularly in T cells, remains elusive.

**Methods:**

In this study, genetic tools were used to edit the human T lymphocyte cell line Jurkat. The activation of HPK1-null cells, HPK1-wildtype cells and HPK1-kinase-inactive cells was compared through ectopic expression of HPK1 in HPK1 knockout cells or direct HPK1 mutation. Besides genetic validation, a series of compounds that selectively target HPK1 (with or without HPK1-degradation activity) were used to assess the potential scaffold function of HPK1 in regulation of human primary T cell activation and cytotoxic activity.

**Results and conclusion:**

Augmented T-cell receptor (TCR)-induced activation in HPK1-knockout Jurkat cells was inhibited by complementation of wildtype, but not kinase-dead HPK1. HPK1 K46E-knockin and K46*-knockin Jurkat cells showed comparable levels of enhanced TCR-induced activation compared with control HPK1-wildtype Jurkat cells. Similarly, HPK1 kinase inhibitor (Compound 1) and cereblon-based (CRBN-based) HPK1 degrader (Compound 2) elicited similar degrees of maximum TCR-induced activation in primary human peripheral blood T cells. In summary, the results of this study suggested that HPK1 kinase inhibitor may be sufficient for HPK1 targeting in T cell mediated cancer immunotherapy.

## 1 Introduction

Hematopoietic progenitor kinase 1 (HPK1; also termed mitogen-activated protein kinase kinase kinase kinase 1 [MAP4K1]) is a MAP4K family kinase belonging to the sterile 20-like serine/threonine kinase superfamily; it is predominantly expressed in hematopoietic cells after the embryonic stage (1, 2). HPK1 is an intracellular immunosuppressive regulator constraining the activation of various immune cells, particularly T cells. Upon T-cell receptor (TCR) engagement, HPK1 is recruited to the complex by adaptor proteins (3, 4), autophosphorylated, and transphosphorylated to achieve full kinase activation (5). Activated HPK1 phosphorylates S376 of SH2 domain containing leukocyte protein of 76kDa (SLP76-S376) to enable interaction of SLP76 with the negative regulator 14-3-3 (6). SLP76-S376 phosphorylation induces ubiquitination and subsequent proteasomal degradation of SLP76, which in turn initiates the destabilization of the TCR signaling complex. Consequently, it attenuates the activating signal transduction downstream of TCR, including the phosphorylation of phospholipase C gamma 1 (PLCγ1) and extracellular signal-regulated kinase (ERK) (7–9). It has also been reported that HPK1 negatively regulates B-cell receptor signaling and dendritic cell activation (10, 11), as well as T-cell and B-cell adhesion (1, 2, 12–15).

HPK1-knockout mice and kinase-dead (KD) HPK1-knockin mice showed an augmented immune response against tumor growth. The CD8^+^ T cell-intrinsic negative regulatory role of HPK1 was validated by adoptive transfer of HPK1 KD-knockin OTI cells in murine tumor models (4, 16). Considering the dominant role of cytotoxic T cells in tumor cell killing, these findings identify HPK1 as a promising target for cancer immunotherapy.

Thus far, hundreds of small molecule inhibitors have been designed to target the kinase function of HPK1, and many of them have shown good efficacy in tumor models (17). At least nine of these inhibitors are under investigation in phase I or phase II clinical studies involving patients with cancer (e.g., CFI-402411, NDI-101150, GRC-54276, BGB-15025, PRJ1-3024, FB-849, BB-3008, RGT-264, and BGB-26808), either as monotherapy or in combination with immunotherapy, such as programmed cell death 1/programmed cell death-ligand 1 (PD-1/PD-L1) blockade.

However, previous reports suggested that HPK1 has complex functions beyond its kinase activity. It has been reported that the C-terminal domain of HPK1 (HPK1-C) generated by caspase 3 (CASP3) cleavage regulates the nuclear factor-κB (NF-κB) pathway and promotes activation-induced cell death (AICD). Guided by these observations, HPK1-targeting degraders were designed to improve tumor suppression. Several cereblon-based (CRBN-based) HPK1 degraders, including SS47 (18), DD205-291 (19) and P1 (20), were evaluated preclinically in comparison with HPK1 kinase inhibitors. Compared to the corresponding warhead, SS47 and P1 demonstrated greater tumor suppression, while DD205-291 exhibited similar tumor suppression. However, the warheads of SS47 and P1 showed poor selectivity, which might impede the analysis of HPK1’s non-catalytic function in antitumor immunity. These findings raise the following question: Is abolition of HPK1 kinase activity sufficient to alleviate the immunosuppressive function of HPK1, particularly in human T cells? The answer to this question may contribute to the rational selection of a HPK1-targeting strategy.

In this study, we utilized genetic tools to evaluate the T cell-inhibitory function of HPK1 KD using the human T lymphocyte cell line Jurkat. We also developed a CRBN-based HPK1 degrader and its related HPK1 inhibitors to selectively target HPK1 in human primary T cells.

## 2 Methods

### 2.1 Cell line and cell culture

Jurkat cells (ATCC, Manassas, VA, USA) and human peripheral blood mononuclear cells (PBMCs) were cultured in RPMI-1640 medium (Thermo Fisher Scientific, Waltham, MA, USA) supplemented with 10% heat-inactivated fetal bovine serum (Thermo Fisher) and 1% penicillin–streptomycin (Thermo Fisher Scientific) at 37°C in a humidified incubator with 5% CO_2_. The density of Jurkat cells was adjusted to 1 × 10^6^/mL 24 h before conducting the experiment. Cryopreserved human PBMCs were purchased from Oribiotech (Shanghai, China); they were freshly thawed and cultured overnight prior to performing the experiment.

The Jurkat line with a homozygous knockout of HPK1 (HKO) single clone was purchased from EdiGene (Beijing, China). HKO-EV (HKO empty vector control), HKO-HPK1.WT (HKO overexpressing wildtype HPK1), and HKO-HPK1.K46E (HKO overexpressing kinase-dead HPK1) Jurkat pools were generated by lenti-vector transduction. In brief, the coding sequences of different HPK1 mutants were inserted into a FG12-BSD plasmid with the human phosphoglycerate kinase (hPGK) promoter (GenScript, Nanjing, China). Thereafter, 293T cells (ATCC) were transfected with those plasmids together with lentivirus packing plasmids pMD2.G and psPAX2. The culture medium containing virus was collected and used to infect HKO cells. Blasticidin 10 µg/mL (InvivoGen, San Diego, CA, USA) was used for the selection of infected cells. HPK1-knockin Jurkat cells (HPK1 K46*) and kinase-inactivated HPK1-knockin Jurkat cells (HPK1 K46E) were produced by introducing a Lys46Ter mutation or a Lys46Glu mutation to the HPK1 gene, respectively.

### 2.2 Western blotting analysis

Jurkat cells were activated by anti-human CD3 (OKT3; 10 μg/mL, Thermo Fisher Scientific), while human peripheral blood cells were activated through TCR crosslinking by biotinylated OKT3 and streptavidin (7.5 μg/mL; Thermo Fisher Scientific). The cells were lysed at various time points post stimulation, and the lysate was denatured. The obtained samples were subjected to electrophoresis, and the separated proteins were electro-transferred onto a nitrocellulose membrane. Thereafter, the membranes were incubated with antibodies against HPK1 (E1C3L), phosphorylated SLP76-S376 (p-SLP76-S376; D7S1K), SLP76 (D1R1A), p-ERK1/2-T202/Y204 (D13.14.4E), p-PLCγ1-Y783 (polyclonal), PLCγ1 (D9H10), and glyceraldehyde-3-phosphate dehydrogenase (GAPDH; D4C6R). All antibodies used for immunoblotting were purchased from Cell Signaling Technology (Danvers, MA, USA).

### 2.3 Intracellular staining and flow cytometry analysis

Jurkat cells or human PBMCs were pretreated in triplicates or quadruplicates with dimethyl sulfoxide or compounds for 24 h. For p-SLP76-S376 analysis, the cells were activated for 30 min with OKT3. For granzyme B and perforin analysis of human PBMCs, ultra-high binding 96-well plates (Corning Inc., Corning, NY, USA) were precoated with OKT3 overnight at 4°C. Human PBMCs were pretreated in quadruplicates with dimethyl sulfoxide or compounds for 24 h. The cells were then activated for another 24 h with precoated OKT3, followed by 2 h treatment with Protein Transport Inhibitor Cocktail (Thermo Fisher). The stimulated cells were fixed with BD Phosflow™ Lyse/Fix buffer (BD Biosciences, San Diego, CA, USA), washed and permeabilized with BD Perm Buffer III (BD Biosciences), and subsequently stained with a fluorescence-conjugated antibody against HPK1 (E1C3L; Cell Signaling Technology), p-SLP76-S376 (E3G9U; Cell Signaling Technology), granzyme B (GB11, BioLegend, San Diego, CA, USA), perforin (dG9, BioLegend) and anti-human CD3 (SK7, BioLegend), CD4 (SK3, BioLegend) and CD8 (SK1, BioLegend) for primary T cells. This was followed by data acquisition and analysis with the BD FACSymphony™ A1 Cell Analyzer (BD Biosciences).

### 2.4 Cytokine analysis

Jurkat cells were seeded in triplicates into those plates for stimulation and incubated for 24 h. Alternatively, Jurkat cells were pretreated with various concentrations of the aforementioned compounds for 24 h prior to stimulation with aforementioned immobilized OKT3. For cytokine analysis of human PBMCs, the cells were seeded into U-bottomed 96-well plates (Corning Inc.) and treated with the compounds for 24 h prior to stimulation. Next, the cells were stimulated by co-culture with HEK293/OS8/PD-L1 cells in the U-bottomed 96-well plates for another 24 h. HEK293/OS8/PD-L1 is a HEK293 cell line edited to stably co-express human PD-L1 (programmed death ligand 1) and single chain fragment variable (scFv) of OKT3 fused with transmembrane and cytoplasmic domains of murine CD8α, as previously described (21). Interleukin 2 (IL-2) and interferon-gamma (IFN-γ) in the supernatant were detected using the HTRF Human IL-2 Detection Kit and AlphaLISA Biotin-Free Human IFNγ Detection Kit (both obtained from Revvity, Waltham, MA, USA) according to the instructions provided by the manufacturer. Data were acquired with PHERAstar FSX (BMG LABTECH Inc., Cary, NC, USA).

### 2.5 Activation-induced cell death assays

For TCR-mediated apoptosis analysis, Jurkat cells were stimulated with aforementioned immobilized OKT3 for 24 h and stained with Annexin V Apoptosis Detection Kit with propidium iodide (PI) (BioLegend) according to the instructions provided by the manufacturer. The cells were analyzed with a BD FACSymphony™ A1 Cell Analyzer (BD Biosciences).

### 2.6 Real-time cell mediated cytotoxicity assay

For *in vitro* HepG2 cell killing, human PBMCs were pretreated in quadruplicates with dimethyl sulfoxide or compounds for 24 h, followed by stimulation by OKT3 for another 24 h. HepG2 cells were seeded at 2 × 10^4^/well in a RTCA 96-well-E plate (Agilent Technologies, Sana Clara, CA, USA). 24 h after HepG2 seeding, the stimulated PBMCs were added at a PBMC: HepG2 cells ratio of 5:1. Target cell death was monitored using xCELLigence RTCA MP instrument (Agilent Technologies).

### 2.7 Statistical analysis

All results were plotted as the mean ± standard error of the mean. Student’s *t*-test was carried out using GraphPad Prism (GraphPad Software, La Jolla, CA, USA) and p-value < 0.05 indicates a statistically significant difference.

## 3 Results

### 3.1 TCR-induced activation of Jurkat cells was augmented in the absence of HPK1

**Figure 1:**
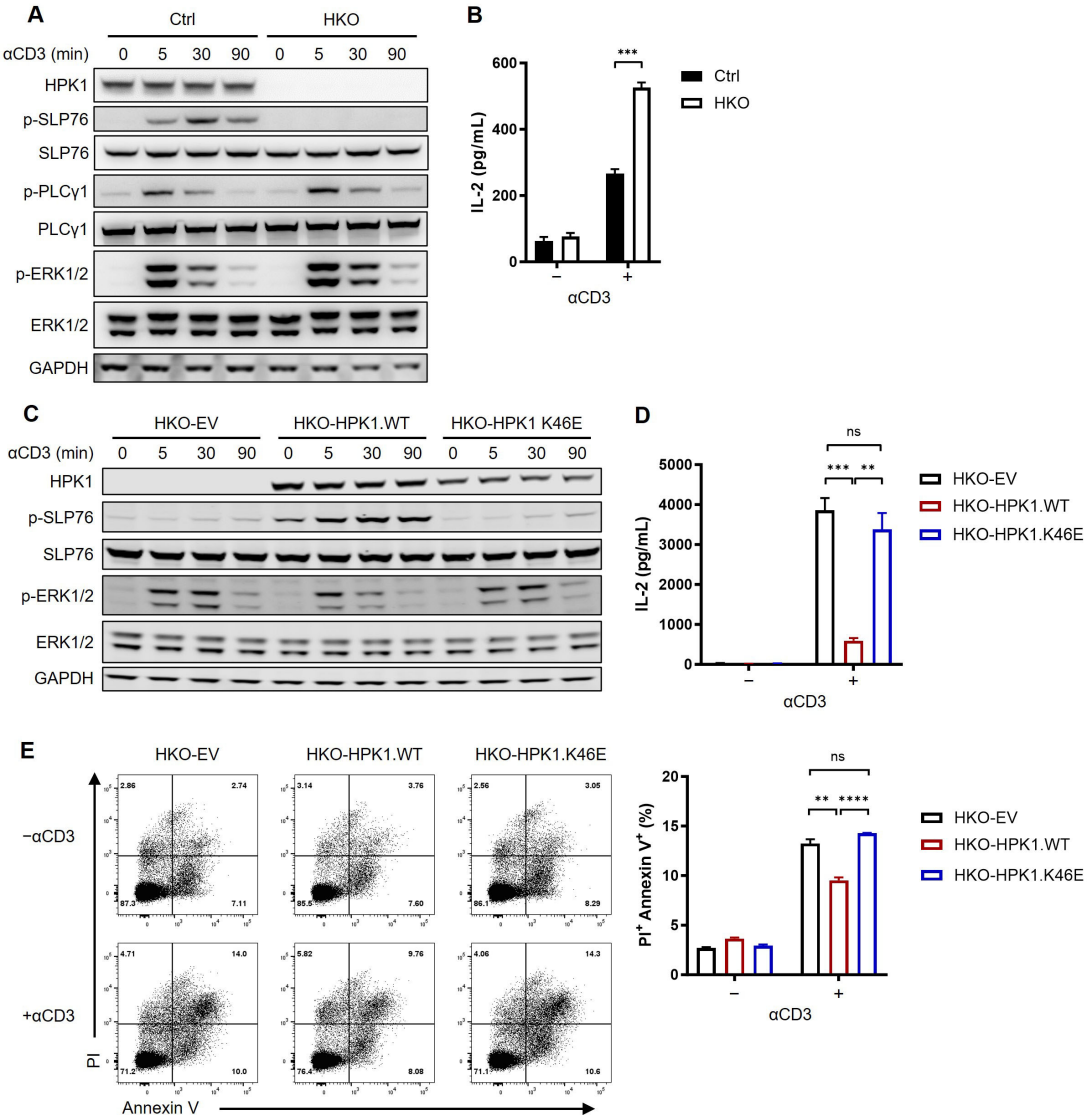
Complementation of a kinase-dead HPK1 failed to inhibit TCR activation in HPK1-knockout Jurkat cells. **(A)** Western blotting analysis of TCR-induced phosphorylation of SLP76-S473, PLCy1, and ERK1/2 in control and HKO Jurkat cells in response to treatment with OKT3. **(B)** IL-2 production in response to stimulation with immobilized OKT3 for 24 h. **(C)** Western blotting analysis of TCR-induced phosphorylation of SLP76-S473 and ERK1/2 in HKO-EV, HKO-HPK1.WT, and HKO- HPK1.K46E Jurkat cells in response to treatment with OKT3. **(D)** IL-2 production in response to stimulation with immobilized OKT3 for 24 h. **(E)** Representative FACS plots and bar graphs of activation-induced cell death in response to stimulation with immobilized OKT3 for 24 h. Data are representative of at least three independent experiments. In each panel, ns, non-significant, **p< 0.01, ***p < 0.001 and ****p < 0.0001. Ctrl, control; ERK1/2, extracellular signal-regulated kinase 1/2; EV, empty vector; FACS, fluorescence-activated cell sorting; GAPDH, glyceraldehyde-3-phosphate dehydrogenase; HKO, knockout of HPK1; HPK1, hematopoietic progenitor kinase 1; IL-2, interleukin-2; ns, not significant; p-, phosphorylated; Pl, propidium iodide; PLCy1, phospholipase C gamma 1; SLP76, SH2 domain containing leukocyte protein of 76kDa; TCR, T-cell receptor; WT, wildtype.

To validate the role of HPK1 in the negative regulation of TCR signaling in Jurkat cells, a Jurkat cell line with a homozygous knockout of HPK1 (HKO) was generated using the clustered regularly interspaced palindromic repeats/CRISPR-associated protein 9 (CRISPR/Cas9) system (**Figure 1A**; **Supplementary Figures S1A, B**). During treatment with the hCD3 antibody OKT3, control Jurkat cells showed significant phosphorylation on S376 of SLP76. In contrast, HKO Jurkat cells exhibited nearly undetectable SLP76-S376 phosphorylation, confirming the previously reported dependence of SLP76-S376 phosphorylation on HPK1 (6). Consistent with previous findings in HPK1 knockout murine primary T cells (8), TCR-induced phosphorylation of PLCγ1 and ERK1/2 were significantly increased and sustained in HKO Jurkat cells compared with control Jurkat cells (**Figure 1A**). Activation of the ERK mitogen-activated protein kinase (MAPK) pathway results in the formation of the AP-1 complex and, in turn, induces IL-2 transcription (22). To evaluate the regulatory role of HPK1 in TCR-induced cytokine production by Jurkat cells, we treated control and HKO Jurkat cells with immobilized OKT3. Consistent with the undetectable SLP76-S376 phosphorylation and increased ERK1/2 phosphorylation, HKO Jurkat cells showed a significant increase in IL-2 production in response to treatment with immobilized OKT3 versus control Jurkat cells (**Figure 1B**). These data confirmed the findings of previous reports on the negative regulatory role of HPK1 in TCR signaling and TCR-induced cytokine production by T cells.

### 3.2 Kinase-inactivated HPK1-complemented Jurkat cells showed comparable augmented activation to that observed for HPK1-deficient Jurkat cells

We sought to further evaluate the role of scaffolding and kinase-related functions of HPK1 in TCR-induced Jurkat activation. Therefore, we generated two Jurkat lines expressing wildtype (WT) HPK1 (HKO-HPK1.WT) and HPK1 with a K46E KD mutation (HKO-HPK1.K46E) based on a HKO Jurkat cell single clone (**Supplementary Figure S1A**). Stimulation with OKT3 resulted in a significant increase in the phosphorylation of SLP76-S376 in HKO-HPK1.WT Jurkat cells. However, there was no increase observed in the phosphorylation of SLP76-S376 in HKO-HPK1.K46E Jurkat cells. This finding was consistent with the results obtained in HKO-EV Jurkat cells (**Figure 1C**). These results further confirmed the non-redundant role of HPK1 kinase activity in SLP76-S376 phosphorylation. HKO-HPK1.K46E and HKO-EV Jurkat cells also showed identically sustained induction of ERK1/2 phosphorylation (up to 30 min) compared with HKO-HPK1.WT Jurkat cells (**Figure 1C**). Consistently, HKO-HPK1.K46E and HKO-EV Jurkat cells produced similar elevated levels of IL-2 in response to treatment with immobilized OKT3 (**Figure 1D**). We utilized the annexin V/PI assay to address the role of HPK1 kinase activity in TCR-induced apoptosis in Jurkat cells treated with immobilized OKT3. After 24 hours of stimulation with OKT3, the percentages of PI^+^ Annexin V^+^ cells in HKO-EV and HKO-HPK1.K46E Jurkat cells were both approximately 1.5-fold higher than that detected in HKO-HPK1.WT Jurkat cells (**Figures 1E, F**), which was consistent to the IL-2 production and TCR signaling data. These results indicated that the kinase-related function of HPK1, rather than its scaffolding function, is critical in the negative regulation of TCR-induced activation of Jurkat cells.

### 3.3 N-terminal truncated HPK1-knockin and HPK1 KD-knockin Jurkat cells showed similar enhanced TCR-induced activation

**Figure 2:**
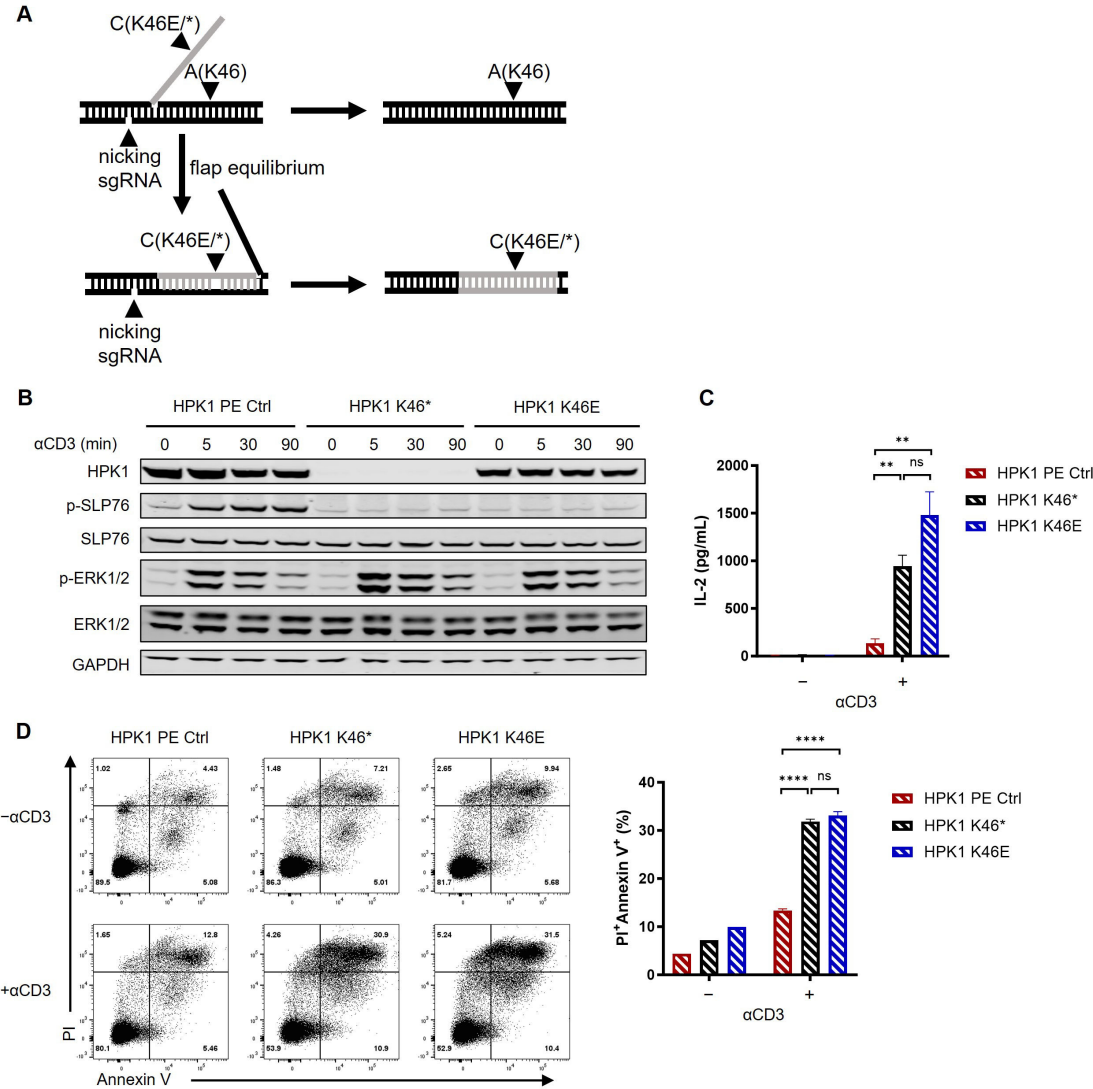
Kinase-dead HPK1 knockin enhanced Jurkat cell activation in a similar manner to HPK1 knockout. **(A)** Schematic of knockin workflow of HPK1 K46* or K46E. **(B)** Western blotting analysis of TCR-induced phosphorylation of SLP76-S473 and ERK1/2 in HPK1 PE Ctrl, HPK1 K46*, and HPK1 K46E Jurkat cells in response to treatment with OKT3. **(C)** IL-2 production in response to stimulation with immobilized OKT3 for 24 h. **(D)** Representative FACS plots and bar graphs of activation-induced cell death in response to stimulation with immobilized OKT3 for 24 h. Data are representative of at least three independent experiments. In each panel, ns, non-significant, **p< 0.01 and ****p < 0.0001. Ctrl, control; ERK1/2, extracellular signal-regulated kinase 1/2; FACS, fluorescence-activated cell sorting; GAPDH, glyceraldehyde-3-phosphate dehydrogenase; HPK1, hematopoietic progenitor kinase 1; IL-2, interleukin-2; ns, not significant; p-, phosphorylated; PE, prime editing; Pl, propidium iodide; SLP76, SH2 domain containing leukocyte protein of 76kDa; sgRNA, single- guide RNA; TCR, T-cell receptor.

We aimed to circumvent any potential interference on the results obtained from our sequential editing of the Jurkat genome. For this purpose, we generated a premature terminated HPK1-knockin Jurkat line (HPK1 K46*) and a kinase-inactivated HPK1-knockin Jurkat line (HPK1 K46E) by introducing a Lys46Ter mutation or a Lys46Glu mutation to the HPK1 gene, respectively (**Figure 2A**).

In previous research, ~50% reduction in HPK1 protein levels was observed in T cells of HPK1 K46E-knockin mice (23). Consistently, in this study, HPK1 K46E knockin in Jurkat cells also resulted in a significant reduction in the levels of HPK1 protein (**Figure 2A**). Despite expression of a full-length mutant HPK1, lack of significant phosphorylation of SLP76-S376 and slower attenuation of ERK1/2 phosphorylation in response to stimulation with OKT3 was observed in HPK1 K46E Jurkat cells; nearly identical findings were obtained for HPK1 K46* Jurkat cells (**Figure 2B**). HPK1 K46* and HPK1 K46E Jurkat cells treated with immobilized OKT3 also showed similar enhanced IL-2 production (**Figure 2C**) and TCR-induced apoptosis (**Figures 2D**). These observations reconfirmed the critical kinase activity and limited scaffolding function of HPK1 in constraining TCR-induced activation.

### 3.4 HPK1-targeting degrader and inhibitors enhanced Jurkat activation

**Figure 3:**
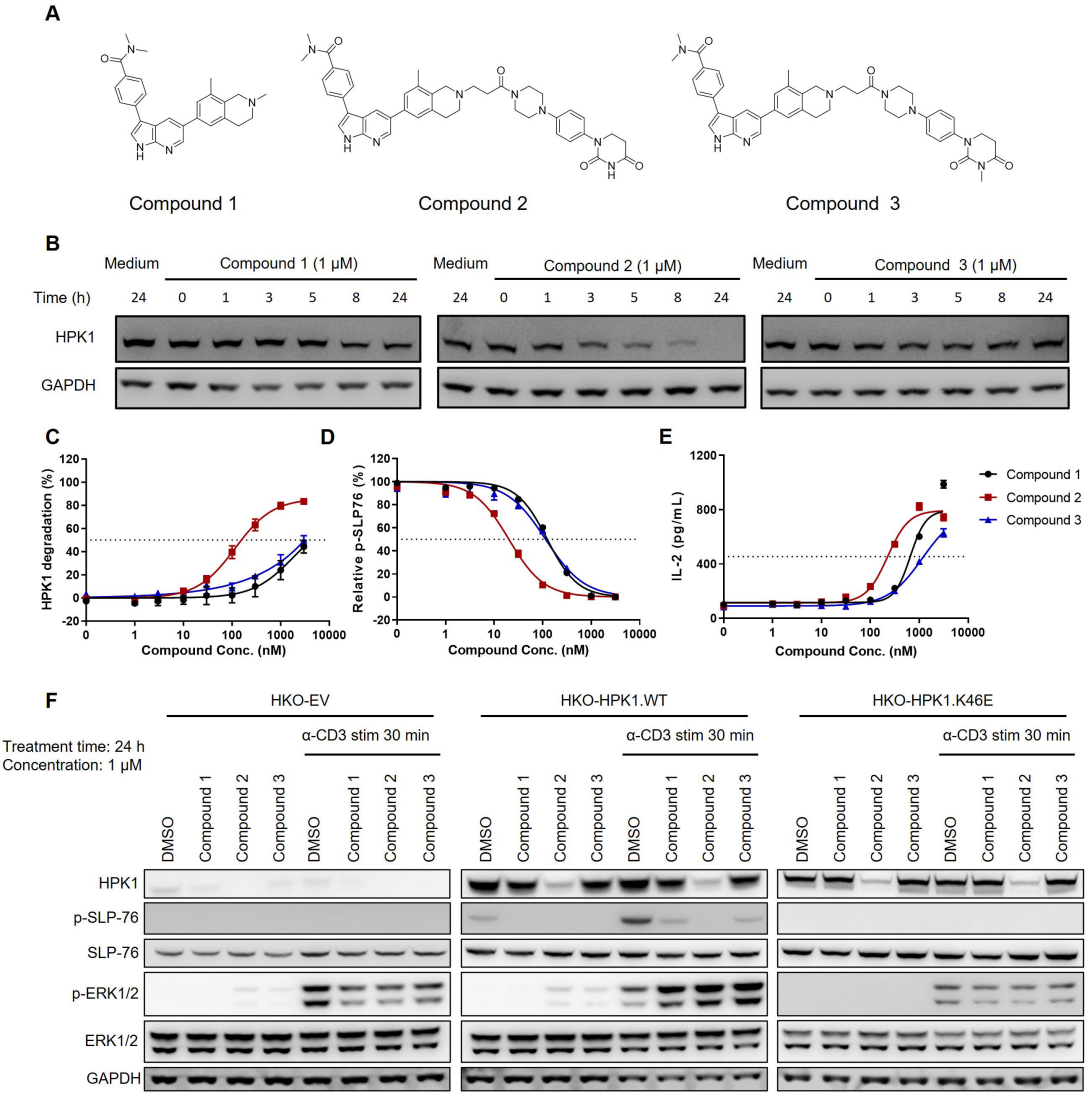
Activity of HPK1 kinase inhibitor and HPK1 degrader. **(A)** Structure of Compound 1, Compound 2, and Compound 3. **(B)** Time course analysis of HPK1 degradation in Jurkat cells pretreated with the indicated compounds. **(C)** HPK1 degradation curve in Jurkat cells upon treatment with the indicated compounds. **(D)** p-SLP76-S473 in Jurkat cells pretreated with the indicated compounds in response to stimulation with OKT3 for 30 min. **(E)** IL-2 production in Jurkat cells pretreated with the indicated compounds in response to stimulation with immobilized OKT3 for 24 h. **(F)** Western blotting analysis of TCR-induced phosphorylation of SLP76-S473 and ERK1/2 in Jurkat cells pretreated with the indicated compounds in response to treatment with OKT3. Data are representative of at least two independent experiments. DMSO, dimethyl sulfoxide; ERK1/2, extracellular signal-regulated kinase 1/2; GAPDH, glyceraldehyde-3-phosphate dehydrogenase; HPK1, hematopoietic progenitor kinase 1; IL-2, interleukin-2; p-, phosphorylated; SLP76, SH2 domain containing leukocyte protein of 76kDa; stim, stimulation; TCR, T-cell receptor; WT, wildtype.

To further examine the potential noncatalytic functions of HPK1 during TCR-induced T-cell activation, we developed a series of compounds that selectively target HPK1, with or without HPK1 degradation activity (**Figure 3A**). These compounds include Compound 2 (a CRBN-based HPK1 degrader), Compound 3 (a related E3-dysfunctional compound) and Compound 1 (a related warhead).

At the concentration of 1 μM, Compound 2 effectively degraded HPK1 in Jurkat cells within 24 h (**Figure 3B**). Treatment of Jurkat cells with the compounds for 24 h revealed a concentration-dependent degradation of HPK1 by Compound 2, with a half maximal effective concentration (EC_50_) of approximately 120 nM. Notably, treatment with Compound 1 and Compound 3 at high concentrations (i.e., ≥1,000 nM) also resulted in the degradation of HPK1 in Jurkat cells (**Figure 3C**), which might be due to the instability of the HPK1–compound complex. All three compounds reduced TCR-induced phosphorylation of SLP76-S376. The calculated half maximal inhibitory concentration (IC_50_) for Compound 2 was approximately 20 nM, while that for Compound 1 and Compound 3 was 120 nM (**Figure 3D**). These results revealed that, at an appropriate concentration, Compound 1 and Compound 3 can effectively inhibit HPK1 kinase activity without significant HPK1 degradation. An approximately 50% reduction in the levels of HPK1 protein and unaffected TCR signaling were noted in T cells of mice with a heterozygous deletion of HPK1 (23). These results suggested that inhibition of HPK1 kinase activity, other than partial degradation of HPK1 protein, was responsible for the reduced SLP76-S376 phosphorylation in Jurkat cells treated with Compound 1 and Compound 3. Pretreatment with Compound 2 for 24 h resulted in enhanced IL-2 production by OKT3-stimulated Jurkat cells, with an EC_50_ of approximately 200 nM (**Figure 3E**). Pretreatment with Compound 1 and Compound 3 also augmented IL-2 production by Jurkat cells to a maximal level comparable with that induced by Compound 2; however, the EC_50_ was higher (**Figure 3E**).

To further validate whether these compounds enhance Jurkat activation in an HPK1-dependent manner, we analyzed TCR-induced signaling in HKO-EV, HKO-HPK1.WT and HKO-HPK1.K46E Jurkat cells after pretreatment with Compound 1, Compound 2 or Compound 3 and stimulation with OKT3. HKO-HPK1.WT Jurkat cells treated with Compound 1, Compound 2 or Compound 3 showed attenuated SLP76-S376 phosphorylation. This evidence indicated effective HPK1 degradation or inhibition of HPK1 kinase activity by the compounds. Treatment with the compounds did not induce SLP76-S376 phosphorylation in HKO-EV and HKO-HPK1.K46E Jurkat cells, reflecting the absence of HPK1 protein (in HKO-EV) or the loss of HPK1 kinase activity (in HKO-HPK1.K46E). Treatment with Compound 1, Compound 2 or Compound 3 enhanced ERK1/2 phosphorylation only in HKO-HPK1.WT Jurkat cells, but not in HKO-EV or HKO-HPK1.K46E Jurkat cells. These data indicated that compound-induced augmentation of ERK1/2 phosphorylation was due to HPK1 targeting rather than an off-target effect (**Figure 3F**).

### 3.5 HPK1 kinase activity controlled TCR-induced activation and cytotoxic activity of human primary T cells

**Figure 4:**
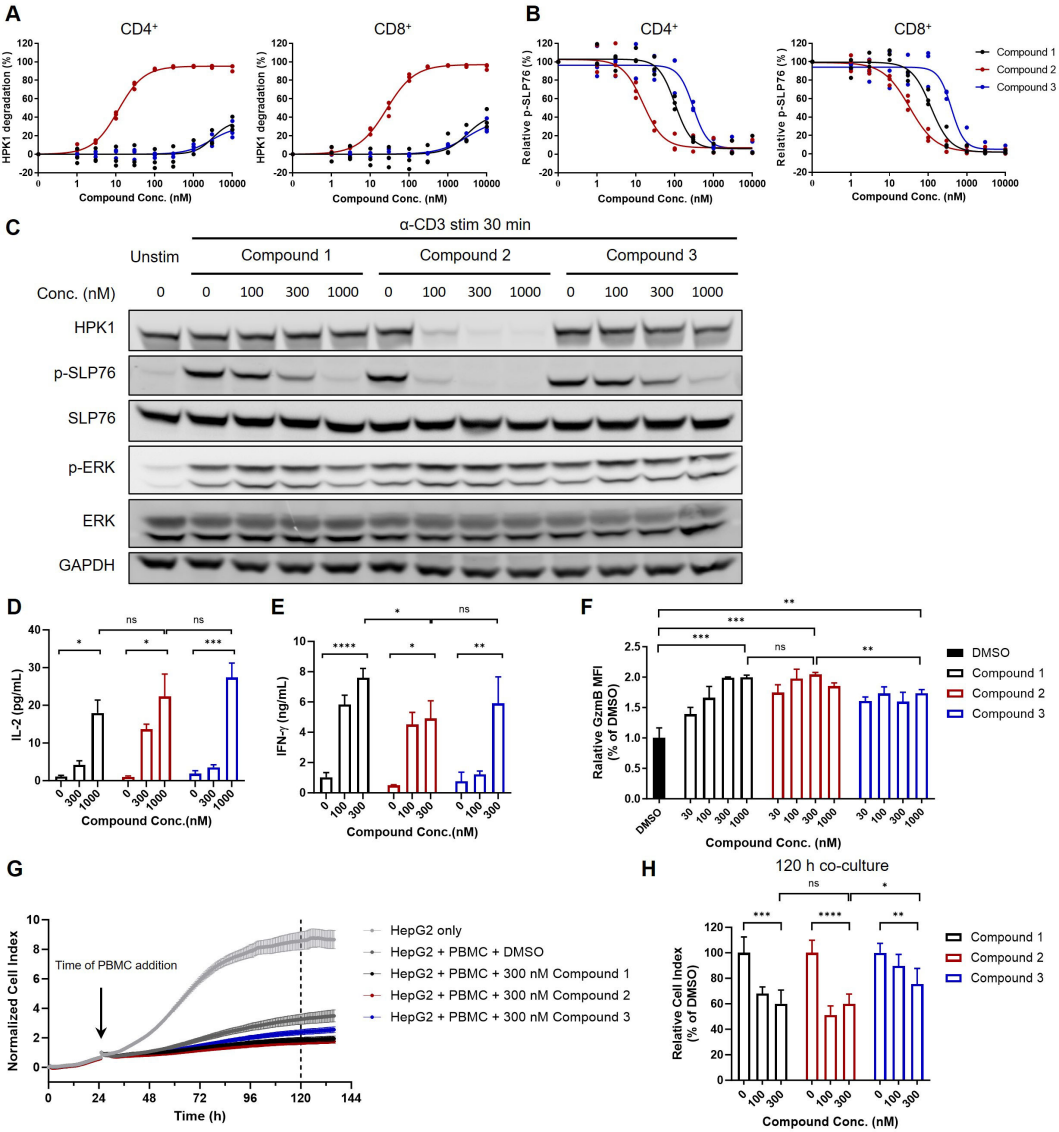
The extent of T cell activation and cytotoxic activity induced by HPK1 kinase inhibitor was similar to that observed with HPK1 degrader in primary human T cells. **(A)** HPK1 degradation curve and **(B)** p-SLP76-S473 inhibition curve for human peripheral CD4+ and CD8+ T cells pretreated with the indicated compounds for 24 h. PBMC samples obtained from three healthy donors were analyzed. **(C)** Western blotting analysis of TCR-induced phosphorylation of SLP76-S473 and ERK1/2 in human PBMC pretreated with compounds in response to treatment with OKT3. Data are representative of two independent experiments using PBMCs obtained from one donor. **(D)** IL-2 and **(E)** IFN-y production in human PBMC pretreated with the indicated compounds after co-culture with HEK293/OS8/PD- L1 for 24 h. Data are representative of four independent experiments using PBMCs obtained from one donor. **(F)** Granzyme B levels in CD8+ T cells of human PBMC pretreated with the indicated compounds followed by OKT3 stimulation for 24 h. Data are representative of three independent experiments using PBMCs obtained from one donor. **(G)** Normalized cell index and **(H)** relative cell index of HepG2 co-cultured with PBMC pretreated with the indicated compounds followed by OKT3 stimulation. Data are representative of three independent experiments using PBMCs obtained from one donor. In each panel, ns, non-significant, *p<0.05, **p< 0.01, ***p < 0.001 and ****p < 0.0001. Conc., concentration; DMSO, dimethyl sulfoxide; ERK1/2, extracellular signal-regulated kinase 1/2; GAPDH, glyceraldehyde-3-phosphate dehydrogenase; GzmB, granzyme B; HPK1, hematopoietic progenitor kinase 1; IFN-y, interferon- gamma; IL-2, interleukin-2; p-, phosphorylated; PBMC, peripheral blood mononuclear cell; PD-L1, programmed cell death-ligand 1; SLP76, SH2 domain containing leukocyte protein of 76kDa; stim, stimulation; TCR, T-cell receptor; Unstim, unstimulated.

Compound 1, Compound 2 and Compound 3 were subsequently used to further investigate the role of the scaffolding and kinase-related functions of HPK1 in TCR-induced activation of human primary T cells. To validate the effectiveness of the compounds in human primary T cells, PBMCs obtained from healthy donors were treated with Compound 1, Compound 2 or Compound 3 for 24 h. This was followed by evaluation of HPK1 degradation and TCR-induced SLP76-S376 phosphorylation in CD4^+^ and CD8^+^ T cells in PBMCs by flow cytometry analysis. Consistent with the effect observed in Jurkat cells, Compound 2 degraded HPK1 in CD4^+^ and CD8^+^ T cells in a concentration-dependent manner (EC_50_ = 11.47 nM and 26.03 nM, respectively). Treatment with Compound 1 or Compound 3 at high concentrations (i.e., ≥3,000 nM) only resulted in limited HPK1 degradation (**Figure 4A**). Compound 1, Compound 2 and Compound 3 effectively reduced TCR-induced phosphorylation on SLP76-S376 in CD4^+^ and CD8^+^ T cells; the IC_50_ was lower in both populations for Compound 2 versus the other two compounds, as in Jurkat cells (**Figure 4B**). Pretreatment with 1,000 nM of Compound 1, Compound 2 or Compound 3 was sufficient to attenuate TCR-induced SLP76-S376 phosphorylation to the background levels, while treatment with 1,000 nM of Compound 1 or Compound 3 did not significantly degrade HPK1.

Next, we analyzed TCR-induced signaling in human PBMCs in response to TCR crosslinking after pretreatment with Compound 1, Compound 2 or Compound 3. Consistent with the observations in CD4^+^ and CD8^+^ T cells, pretreatment with 300 nM of Compound 2 resulted in almost complete degradation of HPK1 and attenuation of SLP76-S376 phosphorylation. Pretreatment with 300 nM and 1,000 nM of Compound 1 or Compound 3 resulted in reduced or almost completely attenuated SLP76-S376 phosphorylation, without significantly affecting HPK1 levels. This attenuation of SLP76-S376 phosphorylation was also associated with a similar increase in ERK phosphorylation (**Figure 4C**). We further tested these compounds in IL-2 (**Figure 4D**) and IFN-γ (**Figure 4E**) production assays in PBMCs. Consistent with the changes in TCR signaling, the maximal enhancement of TCR-induced IL-2 and IFN-γ production by PBMCs after treatment with Compound 2 was not stronger than that after treatment with Compound 1. These results indicated that inhibition of HPK1 kinase activity is sufficient for HPK1 targeting to enhance TCR signaling and effector cytokine production by primary T cells.

We further analyzed the role of HPK1 kinase activity in regulation of T cell cytotoxic activity. Granzyme B is a critical effector molecule produced by activated cytotoxic T cells that induces apoptosis in target cells (24). Pretreatment with Compound 1 or Compound 2 followed by stimulation with OKT3 induced enhanced granzyme B levels in CD8^+^ T cells of human PBMCs in a dose dependent manner, and maximum enhancement induced by Compound 1 and Compound 2 were similar (**Figure 4F**). To directly evaluate the effect of these HPK1-targeting compounds on tumor cell killing activity of primary T cells, we performed real-time HepG2 cell killing assay utilizing the xCELLigence RTCA label-free technology. Consistent with enhancement in granzyme B production, Compound 1 or Compound 2-pretreated PBMCs showed similar increased cytotoxicity against HepG2 cells compared to DMSO-pretreated PBMCs (**Figures 4G, H**). Compound 3 pretreatment also enhanced HepG2 killing ability of PBMCs, but to a less extent than Compound 1 or Compound 2, probably due to poorer permeability of Compound 3 than Compound 1 (**Figures 4G, H**).

Collectively, these findings emphasize the critical kinase activity and limited scaffolding function of HPK1 in the negative regulation of TCR-induced activation and cytotoxic activity in the human primary T cell setting.

## 4 Discussion

The homeostatic balance of the immune system is tightly regulated by positive and negative regulatory circuits within and between immune cells. Cancer immunotherapy provokes anti-tumor immune response by targeting immunosuppressive molecules. HPK1, which has been associated with the negative regulation of T-cell, B-cell, and dendritic-cell functions, is one of the most extensively evaluated immune-oncology targets. However, the complex mechanism underlying the function of HPK1 remains to be further characterized.

Our article mainly focuses on the function of HPK1 in T cells, given their crucial roles in tumor immunity (25, 26). Using HPK1-edited Jurkat cells and HPK1-targeting molecules treated human PBMCs, we evaluated the relative contributions of HPK1’s kinase activity versus non-catalytic function in human T cell settings. We found that the loss of HPK1’s kinase activity and the absence of HPK1 protein led to similar levels of enhancement on TCR-induced activation, cytokine and granzyme B production and cytotoxic activity in human T cells, suggesting the critical role of HPK1’s kinase activity but limited non-catalytic function in these processes.

Some previous studies have highlighted the significance of non-catalytic functions of HPK1 in T cells, while some other literatures suggested that HPK1 kinase inhibition is sufficient for HPK1 targeting in T cells, which is consistent with our findings. The reported non-catalytic functions of HPK1 in T cells can be largely attributed to the C-terminal regulatory domain (HPK1-C) generated by CASP3 during the apoptosis of Jurkat cells, T cell activation, and monocytic differentiation of murine progenitor cells (12, 15, 27). The separated HPK1-N exhibits higher kinase activity than the full-length HPK1, while the separated HPK1-C suppresses NF-ĸB activation through sequestering the inactive IKK complex (15, 27). The continuous activation of human primary T cells shows that with prolonged stimulation, concomitantly with the level of HPK1-C increases, the cells are gradually sensitized towards AICD. In 6-day preactivated T cells, HPK1 knockdown inhibited AICD. The aforementioned article has conducted an in-depth study on the kinetic changes in HPK-C level and the corresponding AICD during T cell activation, and this AICD reduces the tumor-killing activity of T cells by inducing T cell apoptosis. However, since HPK1 regulates T-cell activation via its kinase activity, and AICD is closely related to T-cell activation, it remains uncertain how much impact HPK-C-mediated AICD has on the tumor-killing activity of T cells.

In order to further understand the non-catalytic role of HPK1, we evaluated the relative contribution of HPK1 kinase activity and non-catalytic function in AICD using HPK1-edited Jurkat cell models. Within 24 hours after TCR stimulation, Jurkat cells containing kinase-dead HPK1 (HKO-HPK1.K46E Jurkat cells or HPK1 K46E Jurkat cells) and Jurkat cells devoid of HPK1 (HKO-EV Jurkat cells or HPK1 K46* Jurkat cells) displayed similar enhanced TCR-induced apoptosis. These results indicated limited non-catalytic function of HPK1 in the first 24 hours after TCR stimulation, which is consistent with limited level of HPK1-C at this stage (15). Using the xCELLigence RTCA label-free technology, we were able to monitor cytotoxic activity of HPK1-targeting molecule-pretreated human primary CD8^+^ T cells in PBMCs for up to 6 days post TCR stimulation. However, during this period, Compound 2 (degrader) -pretreated PBMCs did not exhibited stronger cytotoxicity against HepG2 cells compared to Compound 1 (inhibitor) -pretreated PBMCs, indicating that the non-catalytic function of HPK1 is limited at least in regulating the cytotoxic activity of human primary CD8^+^ T cells. Our findings in human T cells were more consistent with previous studies that emphasized HPK1’s critical kinase activity in murine T cell activation: HPK1 knockout and HPK1 KD knockin induce a similar increase in murine T-cell activation (23). Unfortunately, there are currently no additional direct comparative studies published between HPK1 knockout and kinase-dead mice. We also anticipate the literatures associated with this data.

In our study, both genetic validation and compound data support the hypothesis that inhibition of HPK1 kinase activity alone is sufficient for HPK1 targeting in augmenting TCR-induced T-cell activation and cytotoxic activity and to enhance T cell-mediated antitumor immunity. However, our research was conducted under *in vitro* conditions with compound treatment duration of less than 6 days, which may not fully recapitulate the real clinical systems. Therefore, we expect that future comparisons on antitumor activities of HPK1 inhibitors and degraders in clinical trials will provide us insights into the relative contribution of HPK1’s kinase function and non-catalytic function in antitumor immunity, particularly since an HPK1 degrader has already entered the clinical stage (CTR20244285).

## Data Availability

The original contributions presented in the study are included in the article/**Supplementary Materials**. Further inquiries can be directed to the corresponding author.
