# Supplementary figures and images for: HPK1 kinase inhibitor: a sufficient approach to target HPK1 to modulate T cell activation in cancer immunotherapy compared with degraders
Source: Front Immunol. 2025 Feb 6;16:1449106. doi: 10.3389/fimmu.2025.1449106 (PMC11839646; doi:10.3389/fimmu.2025.1449106)

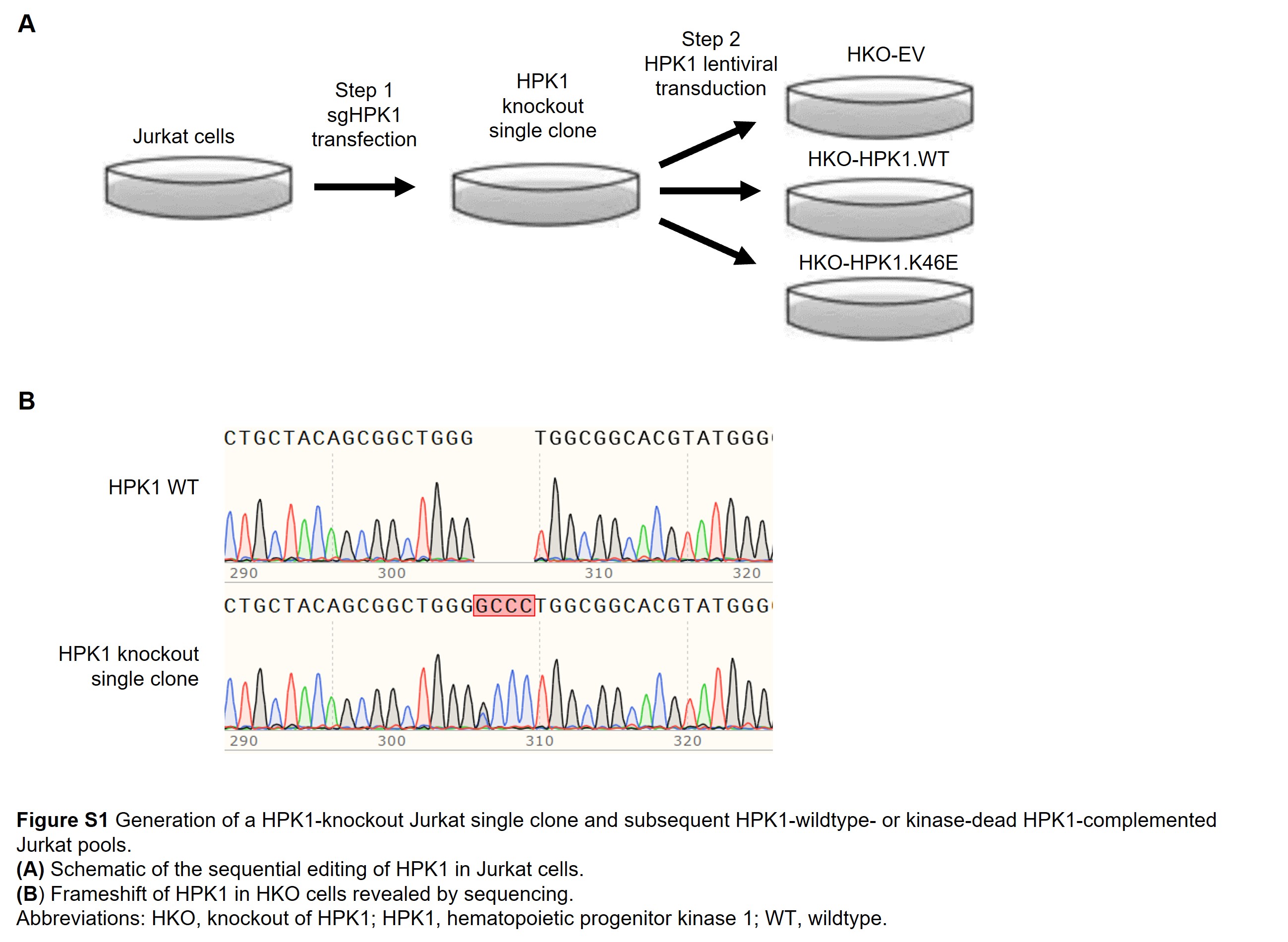

Supplement: Supplementary file 1 [file Image1.jpeg]
