# Supplementary material for: HPK1 kinase inhibitor: a sufficient approach to target HPK1 to modulate T cell activation in cancer immunotherapy compared with degraders
Source: Front Immunol. 2025 Feb 6;16:1449106. doi: 10.3389/fimmu.2025.1449106 (PMC11839646; doi:10.3389/fimmu.2025.1449106)
Supplement: Supplementary file 2 [file Table1.docx]

**Compound Synthesis**

**General Protocol**

Nuclear magnetic resonance (NMR) spectra were recorded on a 400 MHz Varian instrument at an ambient temperature. Chemical shifts are reported in parts per million (δ) using the residual solvent as the internal standard. Liquid chromatography-mass spectrometry (LC-MS) was acquired with a mass spectrometer operating in ES (+) ionization mode. HPLC of Compound 1, Compound 2 and Compound 3 was assayed with >95% purity at 214 nm in a 5.5-minute method. Starting materials were purchased from general vendors or synthesized based on public literatures.

**Synthesis of Compound 1: 4-(5-(2,8-dimethyl-1,2,3,4-tetrahydroisoquinolin-6-yl)-1H-pyrrolo[2,3-b]pyridin-3-yl)-N,N-dimethylbenzamide**

Step 1: 4-(5-bromo-1-tosyl-1H-pyrrolo[2,3-b]pyridin-3-yl)-N,N-dimethylbenzamide

To a solution of 5-bromo-3-iodo-1-tosyl-1H-pyrrolo[2,3-b]pyridine (2.38 g, 5 mmol) in dioxane (30 mL) and water (3 mL) was added (4-(dimethylcarbamoyl)phenyl)boronic acid (965 mg, 5 mmol), K_2_CO_3_ (1.38 g, 10 mmol) and Pd(dppf)Cl_2._CH_2_Cl_2_ (409 mg, 0.5 mmol), and the mixture was stirred for 16 h at 90 ^o^C under N_2_. The mixture was cooled to room temperature, diluted with EtOAc (50 mL), washed with H_2_O (30 mL), brine (30 mL), dried over Na_2_SO_4_, filtered and concentrated under vacuum. The residue was purified by silica gel chromatograph with PE: EtOAc=1:5 to give the title compound (1.50 g, 60%). LC-MS (M+H)^+^ =498.1.

Step 2: N,N-dimethyl-4-(5-(4,4,5,5-tetramethyl-1,3,2-dioxaborolan-2-yl)-1-tosyl-1H-pyrrolo[2,3-b]pyridin-3-yl)benzamide

To a solution of 4-(5-bromo-1-tosyl-1H-pyrrolo[2,3-b]pyridin-3-yl)-N,N-dimethylbenzamide (2.0 g, 4.0 mmol) in dioxane (15 mL) was added BPD (1.52 g, 5.99 mmol), KOAc (1.18 g, 12.0 mmol), Pd(dppf)Cl_2_.CH_2_Cl_2_ (328 mg, 0.401 mmol) at room temperature under nitrogen atmosphere. The resulting mixture was stirred for 3 h at 100 ^o^C. The mixture was cooled to room temperature and diluted with water (50 mL). The mixture was extracted with ethyl acetate (100 mL x 3). The organic phases were combined, washed with brine and dried over Na_2_SO_4_. The solvent was removed under reduced pressure and the residue was purified by flash chromatography eluting with MeOH in DCM (0% to 65 % gradient) to give the title compound (2.04 g, 93 %). LCMS (M+H)^+^ = 546.2.

Step 3: O-pivaloylhydroxylamine trifluoromethanesulfonate salt

TfOH (18.9 g, 125.7 mmol) was added to a solution of tert-butyl (pivaloyloxy)carbamate (24.8 g, 114.3 mmol) in MTBE (230 mL) at 0 °C and stirred at room temperature for 4 h. The volume of solution was reduced to about 100 mL under reduced pressure and the precipitate was collected by filtration. The solid was dried under vacuum to give the title compound (26.0 g, 85%). LC-MS (M+H)^+^ =118.0.

Step 4: 4-bromo-2-methyl-N- (pivaloyloxy)benzamide

DIPEA (15.7 g, 121.6 mmol) was added to a solution of 4-bromo-2-methylbenzoic acid (8.82 g, 40.52 mmol) in THF (150 mL) at 0 °C, followed by T3P (25.8 g, 81.1 mmol) and O-pivaloylhydroxylamine trifluoromethanesulfonate salt (26.0 g, 97.3 mmol). The reaction was stirred at room temperature overnight. Brine (100 mL) was added and the mixture was extracted with ethyl acetate (100 mL x 3). The combined organic layer was dried over Na_2_SO_4_, filtered and concentrated under reduced pressure. The residue was purified by silica gel column chromatography to give the title compound (7.5 g, 59%). LC-MS (M+H)^+^ =314.0.

Step 5: 6-bromo-8-methyl-3, 4-dihydroisoquinolin-1 (2H)-one

KOAc (5.16 g, 52.5 mmol) and dichloro (pentamethylcyclopentadienyl)rhodium (III) dimer (737.7 mg, 1.19 mmol) were added to a solution of 4-bromo-2-methyl-N- (pivaloyloxy)benzamide (7.5 g, 23.9 mmol) in acetonitrile (150 mL). The solution was stirred under an ethylene atmosphere (3 bar) at room temperature for overnight. The solvent was removed in vacuo and the residue was partitioned between water (20 mL) and ethyl acetate (50 mL). The organic layer was dried over Na_2_SO_4_, filtered and concentrated. The residue was purified by silica gel column chromatography to give the title compound (4.67 g, 82%). LC-MS (M+H)^+^ =240.0.

Step 6: 6-bromo-8-methyl-1, 2, 3, 4-tetrahydroisoquinoline

To 6-bromo-8-methyl-3, 4-dihydroisoquinolin-1 (2H)-one (4.67 g, 19.5 mmol) was added BH_3_ in THF (1.0 M, 77.8 mL, 77.8 mmol) and the reaction mixture was refluxed overnight. The mixture was cooled to 0 °C and MeOH (5 mL) was added followed by HCl (2 M, 25 mL). The solution was heated to 80 °C for 3 h. The mixture was cooled to room temperature and solvent was removed in vacuo. The residue was dissolved in DCM (50 mL) and the solution was successively washed with saturated NaHCO_3_ (30 mL) and brine (30 mL), dried over Na_2_SO_4_, filtered and concentrated. The residue was purified by silica gel column chromatography to give the title compound (3.79 g, 86%). LC-MS (M+H)^+^ =226.0.

Step 7: 6-bromo-2,8-dimethyl-1,2,3,4-tetrahydroisoquinoline

Formalin (37%, 3.48 g, 42.9 mmol) was added to a solution of 6-bromo-8-methyl-1, 2, 3, 4-tetrahydroisoquinoline (1.94 g, 8.58 mmol) in DCM (30 mL). After 5 min, NaBH (OAc)_3_ (3.64 g, 17.2 mmol) was added and the mixture was stirred at room temperature overnight. Saturated NaHCO_3_ (20 mL) was added and the mixture was extracted with DCM (30 mL x 2). The combined organic layer was dried over Na_2_SO_4_, filtered and concentrated. The residue was purified by silica gel column chromatography to give the title compound (1.85 g, 90%). LC-MS (M+H)^+^ =240.0.

Step 8: 4-(5-(2,8-dimethyl-1,2,3,4-tetrahydroisoquinolin-6-yl)-1-tosyl-1H-pyrrolo[2,3-b]pyridin-3-yl)-N,N-dimethylbenzamide

To a solution of 6-bromo-2,8-dimethyl-1,2,3,4-tetrahydroisoquinoline (76 mg, 0.316 mmol) in dioxane (8 mL) were added N,N-dimethyl-4-(5-(4,4,5,5-tetramethyl-1,3,2-dioxaborolan-2-yl)-1-tosyl-1H-pyrrolo[2,3-b]pyridin-3-yl)benzamide (207 mg, 0.338 mmol), Pd(dppf)Cl_2_.CH_2_Cl_2_ (38 mg, 0.047 mmol) and a solution of K_2_CO_3_ (110 mg, 0.799 mmol) in H_2_O (2 mL) at room temperature under nitrogen atmosphere. The resulting mixture was stirred for 3 h at 100 ^o^C under nitrogen atmosphere. After the reaction was done, the reaction mixture was concentrated under reduced pressure, and the residue was purified by flash chromatography eluting with MeOH in EtOAc (0 % to 10 % gradient) to give the title compound (68 mg, 54 %). LCMS (M+H)^+^ = 579.4.

Step 9: 4-(5-(2,8-dimethyl-1,2,3,4-tetrahydroisoquinolin-6-yl)-1H-pyrrolo[2,3-b]pyridin-3-yl)-N,N-dimethylbenzamide

To a solution of 4-(5-(2,8-dimethyl-1,2,3,4-tetrahydroisoquinolin-6-yl)-1-tosyl-1H-pyrrolo[2,3-b]pyridin-3-yl)-N,N-dimethylbenzamide (68 mg, 0.12 mmol) in MeOH (5 mL) was added NaOH aqueous solution (0.2 mL, 0.40 mmol, 2M). The resulting mixture was stirred for 1 h at 65 ^o^C. The mixture was cooled to room temperature concentrated under reduced pressure. The residue was purified by prep-HPLC to give Compound 2 (9 mg, 19%). ^1^H NMR (400 MHz, DMSO-*d6*) δ 12.07 (s, 1 H), 8.54 (d, J = 2.0 Hz, 1 H), 8.43 (d, J = 2.0 Hz, 1 H), 8.00 (d, J = 2.8 Hz, 1 H), 7.85 (d, J = 8.4 Hz, 2 H), 7.49 (d, J = 8.4 Hz, 2 H), 7.39 (s, 1 H), 7.35 (s, 1 H), 3.45 (s, 2 H), 3.00 (s, 6 H), 2.96-2.88 (m, 2 H), 2.65-2.58 (m, 2 H), 2.42 (s, 3 H), 2.25 (s, 3 H). LC-MS (M+H)^+^ = 425.6.

**Synthesis of Compound 2: 4-(5-(2-(3-(4-(4-(2,4-dioxotetrahydropyrimidin-1(2H)-yl)phenyl)piperazin-1-yl)-3-oxopropyl)-8-methyl-1,2,3,4-tetrahydroisoquinolin-6-yl)-1H-pyrrolo[2,3-b]pyridin-3-yl)-N,N-dimethylbenzamide**

Step 1: tert-butyl 6-bromo-8-methyl-3,4-dihydro-1H-isoquinoline-2-carboxylate

At 0 ^o^C, to a solution of 6-bromo-8-methyl-1,2,3,4-tetrahydroisoquinoline hydrochloride (36.0 g, 137 mmol) in DCM (400 mL) was added triethylamine (34.7 g, 343 mmol), DMAP (1.67 g, 13.7 mmol) and Boc_2_O (74.8 g, 342.1 mmol) in portions. The mixture was warmed to room temperature and stirred for 16 h. The mixture was concentrated under vacuum. The residue was purified by silica gel chromatography, eluting with EtOAc in PE (0% to 50% gradient) to give the title compound (23.3 g, 52%). LC-MS (M-t-Bu+H)^+^ = 269.9.

Step 2: tert-butyl 8-methyl-6-(4,4,5,5-tetramethyl-1,3,2-dioxaborolan-2-yl)-3,4-dihydro-1H-isoquinoline-2-carboxylate

To a solution of tert-butyl 6-bromo-8-methyl-3,4-dihydro-1H-isoquinoline-2-carboxylate (20.0 g, 61.3 mmol) in dioxane (500 mL) was added BPD (23.4 g, 92.1 mmol), KOAc (18.0 g, 183.9 mmol) and Pd(dppf)Cl_2_.CH_2_Cl_2_ (5.0 g, 6.1 mmol) under nitrogen. The reaction was heated to 100 ^o^C and stirred for 3 h. The mixture was cooled to room temperature and concentrated under reduced pressure. The residue was purified by silica gel chromatography, eluting with EtOAc in PE (0% to 50% gradient) to give the title compound (19.0 g, 83%). LC-MS (M-Boc+H)^+^ = 274.1.

Step 3: tert-butyl 6-(3-(4-(dimethylcarbamoyl)phenyl)-1-tosyl-1H-pyrrolo[2,3-b]pyridin-5-yl)-8-methyl-3,4-dihydroisoquinoline-2(1H)-carboxylate

To a solution of 4-(5-bromo-1-tosyl-1H-pyrrolo[2,3-b]pyridin-3-yl)-N,N-dimethylbenzamide (994 mg, 2 mmol) in dioxane (10 mL) and water (1 mL) were added tert-butyl 8-methyl-6-(4,4,5,5-tetramethyl-1,3,2-dioxaborolan-2-yl)-3,4-dihydro-1H-isoquinoline-2-carboxylate (746 mg, 2 mmol), K_2_CO_3_ (828 mg, 6 mmol) and Pd(dppf)Cl_2._CH_2_Cl_2_ (164 mg, 0.2 mmol), then stirred for 3 h at 100 ^o^C under N_2_. The mixture was cooled to room temperature, diluted with EtOAc (30 mL), washed with H_2_O (20 mL), brine (20 mL), dried over Na_2_SO_4_, filtered and concentrated under vacuum. The residue was purified by silica gel chromatography with EtOAc to give the title compound (1.0 g, 75%). LC-MS (M+H)^+^ =665.4.

Step 4: N,N-dimethyl-4-(5-(8-methyl-1,2,3,4-tetrahydroisoquinolin-6-yl)-1-tosyl-1H-pyrrolo[2,3-b]pyridin-3-yl)benzamide hydrochloride

To a solution of tert-butyl 6-(3-(4-(dimethylcarbamoyl)phenyl)-1-tosyl-1H-pyrrolo[2,3-b]pyridin-5-yl)-8-methyl-3,4-dihydroisoquinoline-2(1H)-carboxylate (1.0 g, 1.5 mmol) in dioxane (2 mL) was added HCl/dioxane (4 M, 10 mL) dropwise at 0 ^o^C. The reaction mixture was stirred for 1 h at room temperature and then concentrated under vacuum to give the title compound (0.90 g, 99%). LC-MS (M+H)^+^ =565.3.

Step 4: tert-butyl 3-(6-(3-(4-(dimethylcarbamoyl)phenyl)-1-tosyl-1H-pyrrolo[2,3-b]pyridin-5-yl)-8-methyl-3,4-dihydroisoquinolin-2(1H)-yl)propanoate

To a solution of N,N-dimethyl-4-(5-(8-methyl-1,2,3,4-tetrahydroisoquinolin-6-yl)-1-tosyl-1H-pyrrolo[2,3-b]pyridin-3-yl)benzamide hydrochloride (400 mg, 0.67 mmol) in DMF (5 mL) was added tert-butyl 3-bromopropanoate (890 mg, 4.26 mmol). The reaction mixture was stirred for overnight at 40 ^o^C, diluted with EtOAc (30 mL), washed with brine (2x 15 mL), dried over Na_2_SO_4_, filtered and concentrated under vacuum. The residue was purified by prep-TLC with MeOH: DCM=1:25 to give the title compound (200 mg, 43%). LC-MS (M+H)^+^ =693.4.

Step 5: tert-butyl 3-(6-(3-(4-(dimethylcarbamoyl)phenyl)-1H-pyrrolo[2,3-b]pyridin-5-yl)-8-methyl-3,4-dihydroisoquinolin-2(1H)-yl)propanoate

To a solution of tert-butyl 3-(6-(3-(4-(dimethylcarbamoyl)phenyl)-1-tosyl-1H-pyrrolo[2,3-b]pyridin-5-yl)-8-methyl-3,4-dihydroisoquinolin-2(1H)-yl)propanoate (200 mg, 0.29 mmol) in dioxane (4 mL) and water (2 mL) was added K_2_CO_3_ (239 mg, 1.73 mmol). The reaction mixture was stirred for 16 h at 100 ^o^C, diluted with EtOAc (20 mL), washed with H_2_O (10 mL), brine (10 mL), dried over Na_2_SO_4_, filtered and concentrated under vacuum. The residue was purified by prep-TLC with MeOH: DCM=1:25 to give the title compound (120 mg, 77%). LC-MS (M+H)^+^ =539.4.

Step 6: 3-(6-(3-(4-(dimethylcarbamoyl)phenyl)-1H-pyrrolo[2,3-b]pyridin-5-yl)-8-methyl-3,4-dihydroisoquinolin-2(1H)-yl)propanoic acid mono trifluoroacetic acid

To a solution of tert-butyl 3-(6-(3-(4-(dimethylcarbamoyl)phenyl)-1H-pyrrolo[2,3-b]pyridin-5-yl)-8-methyl-3,4-dihydroisoquinolin-2(1H)-yl)propanoate (120 mg, 0.22 mmol) in DCM (4 mL) was added TFA (2 mL) dropwise at 0 ^o^C. The reaction mixture was stirred for 1 h at room temperature and then concentrated under vacuum to give the title compound (132 mg, 100%). LC-MS (M+H)^+^ =483.3.

Step 7: 4-(5-(2-(3-(4-(4-(2,4-dioxotetrahydropyrimidin-1(2H)-yl)phenyl)piperazin-1-yl)-3-oxopropyl)-8-methyl-1,2,3,4-tetrahydroisoquinolin-6-yl)-1H-pyrrolo[2,3-b]pyridin-3-yl)-N,N-dimethylbenzamide

To a solution of 3-(6-(3-(4-(dimethylcarbamoyl)phenyl)-1H-pyrrolo[2,3-b]pyridin-5-yl)-8-methyl-3,4-dihydroisoquinolin-2(1H)-yl)propanoic acid mono trifluoroacetic acid (90 mg, 0.15 mmol) in DMF (4 mL) was added Et_3_N (121 mg, 1.2 mmol), EDCI (73 mg, 0.38 mmol), HOBT (51 mg, 0.38 mmol) and 1-(4-(piperazin-1-yl)phenyl)dihydropyrimidine-2,4(1H,3H)-dione hydrochloride (51 mg, 0.19 mmol). The reaction mixture was stirred for overnight at room temperature, diluted with EtOAc (20 mL), washed with brine (2x10 mL), dried over Na_2_SO_4_, filtered and concentrated under vacuum. The residue was purified by prep-TLC with MeOH: DCM=1:8, then purified by prep-HPLC to give compound 2 (20 mg, 18%).^1^H NMR (400 MHz, DMSO-d6) δ 12.07 (s, 1H), 10.28 (s, 1H), 8.54 (s, 1H), 8.42 (s, 1H), 8.02 – 7.98 (m, 1H), 7.87 – 7.82 (m, 2H), 7.52 – 7.47 (m, 2H), 7.39 (s, 1H), 7.34 (s, 1H), 7.19 – 7.14 (m, 2H), 6.99 – 6.93 (m, 2H), 3.71 – 3.65 (m, 4H), 3.64 – 3.61 (m, 2H), 3.58 – 3.53 (m, 2H), 3.19 – 3.14 (m, 2H), 3.12 – 3.08 (m, 2H), 3.01 (s, 6H), 2.97 – 2.91 (m, 2H), 2.87 – 2.81 (m, 2H), 2.74 – 2.66 (m, 6H), 2.27 (s, 3H). LC-MS (M+H)^+^ =739.4.

**Synthesis of Compound 3: N,N-dimethyl-4-(5-(8-methyl-2-(3-(4-(4-(3-methyl-2,4-dioxotetrahydropyrimidin-1(2H)-yl)phenyl)piperazin-1-yl)-3-oxopropyl)-1,2,3,4-tetrahydroisoquinolin-6-yl)-1H-pyrrolo[2,3-b]pyridin-3-yl)benzamide**

To a solution of 3-(6-(3-(4-(dimethylcarbamoyl)phenyl)-1H-pyrrolo[2,3-b]pyridin-5-yl)-8-methyl-3,4-dihydroisoquinolin-2(1H)-yl)propanoic acid mono trifluoroacetic acid (90 mg, 0.15 mmol) in DMF (4 mL) was added Et_3_N (121 mg, 1.2 mmol), EDCI (73 mg, 0.38 mmol), HOBT (51 mg, 0.38 mmol) and 3-methyl-1-(4-(piperazin-1-yl)phenyl)dihydropyrimidine-2,4(1H,3H)-dione hydrochloride (62 mg, 019 mmol). The reaction mixture was stirred for overnight at room temperature, diluted with EtOAc (20 mL), washed with brine (2x10 mL), dried over Na2SO4, filtered and concentrated under vacuum. The residue was purified by prep-TLC with MeOH: DCM=1:8, then purified by prep-HPLC to give compound 3 (20 mg, 18%). ^1^H NMR (400 MHz, DMSO-d6) δ 12.08 (s, 1H), 8.59 – 8.51 (m, 1H), 8.42 (d, J = 1.7 Hz, 1H), 7.99 (s, 1H), 7.84 (d, J = 8.1 Hz, 2H), 7.49 (d, J = 8.1 Hz, 2H), 7.39 (s, 1H), 7.34 (s, 1H), 7.16 (d, J = 8.8 Hz, 2H), 6.95 (d, J = 8.9 Hz, 2H), 3.74 – 3.59 (m, 6H), 3.56 (s, 2H), 3.22 – 3.07 (m, 4H), 3.03 (s, 3H), 3.00 (s, 6H), 2.93 – 2.86 (m, 2H), 2.86 – 2.75 (m, 4H), 2.75 – 2.64 (m, 4H), 2.26 (s, 3H). LC-MS (M+H)^+^ =753.6.
